# Supplementary material for: Modes of Interactions with DNA/HSA Biomolecules and Comparative Cytotoxic Studies of Newly Synthesized Mononuclear Zinc(II) and Heteronuclear Platinum(II)/Zinc(II) Complexes toward Colorectal Cancer Cells
Source: Int J Mol Sci. 2024 Mar 6;25(5):3027. doi: 10.3390/ijms25053027 (PMC10931776; doi:10.3390/ijms25053027)
Supplement: Supplementary file 1 [file ijms-25-03027-s001.zip › ijms-2869146-supplementary.pdf]

Supplementary material

Modes of interactions with DNA/BSA biomolecules and comparative cytotoxic studies of newly synthesized mononuclear zinc(II) and heteronuclear platinum(II)/zinc(II) complexes toward colorectal cancer cells

Samir Vučelj[1,5], Rušid Hasić[2], Darko Ašanin[3], Biljana Šmit[3], Angelina Caković[3], Jovana Bogojeski[3], Marina Ćendić Serafinović[3], Bojana Simović Marković[1], Bojan Stojanović[1,6], Sladjana Pavlović[1], Isidora Stanisavljević[1], Irfan Ćorović[1,5], Milica Dimitrijević Stojanović[1,7], Ivan Jovanović[1], Tanja V. Soldatović[2*], and Bojana Stojanović[1,]

1 Faculty of Medical Sciences, Center for Molecular Medicine and Stem Cell Research, University of Kragujevac, S. Markovića 69, 34000 Kragujevac, Serbia

2 Department of Natural-Mathematical Sciences, State University of Novi Pazar, Vuka Karadžića 9, 36300 Novi Pazar, Serbia;

3 Institute for Information Technologies, University of Kragujevac, Jovana Cvijića bb, Kragujevac 34000, Serbia

4 Faculty of Science, University of Kragujevac, Radoja Domanovića 12, 34000 Kragujevac, Serbia

5 General Hospital of Novi Pazar, Department of Internal Medicine, Generala Živkovića 1, 36300 Novi Pazar

6 Faculty of Medical Sciences, Department of Surgery, University of Kragujevac, S. Markovića 69, 34000 Kragujevac, Serbia

7 Faculty of Medical Sciences, Department of Pathology, University of Kragujevac, S. Markovića 69, 34000 Kragujevac, Serbia

8 Faculty of Medical Sciences, Department of Pathophysiology, University of Kragujevac, S. Markovića 69, 34000 Kragujevac, Serbia

* Correspondence: tsoldatovic@np.ac.r

[ZnCl$_2$(terpy^{tBu})]

a) UV-Vis

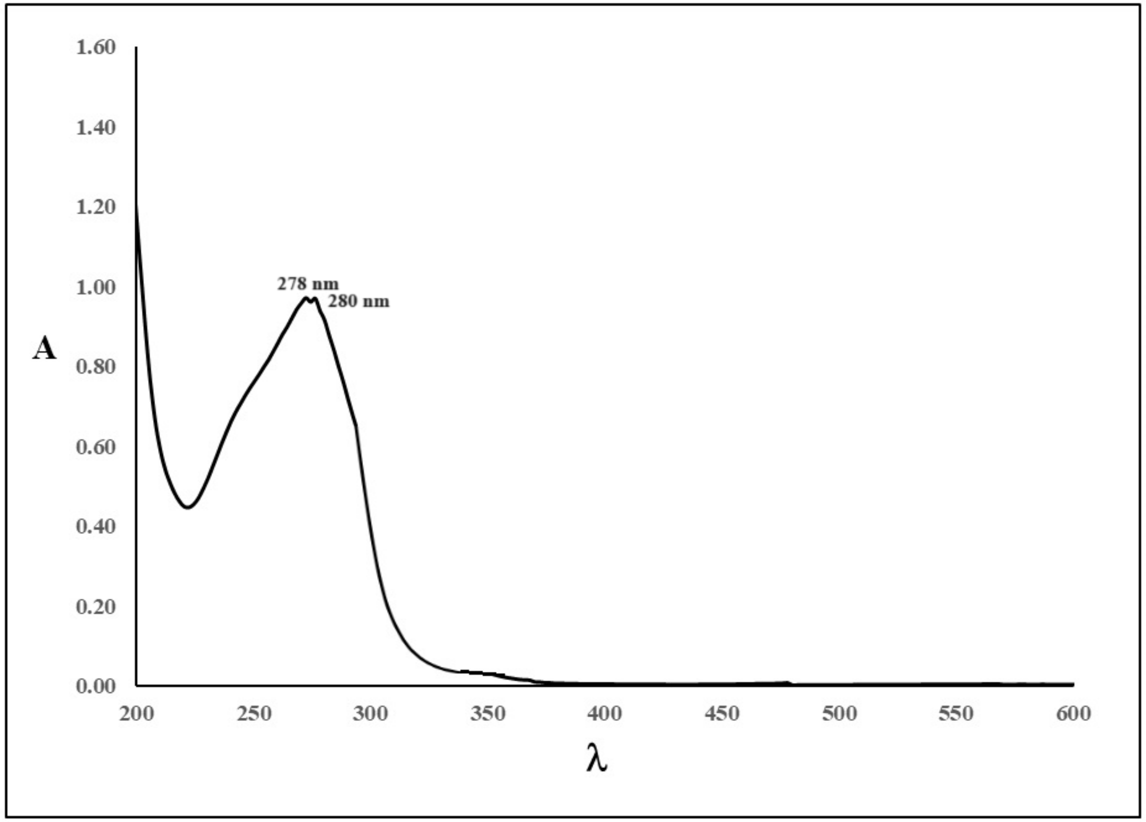

(278, 0.9948) (280, 0.9935)

b) FT-IR (KBr)

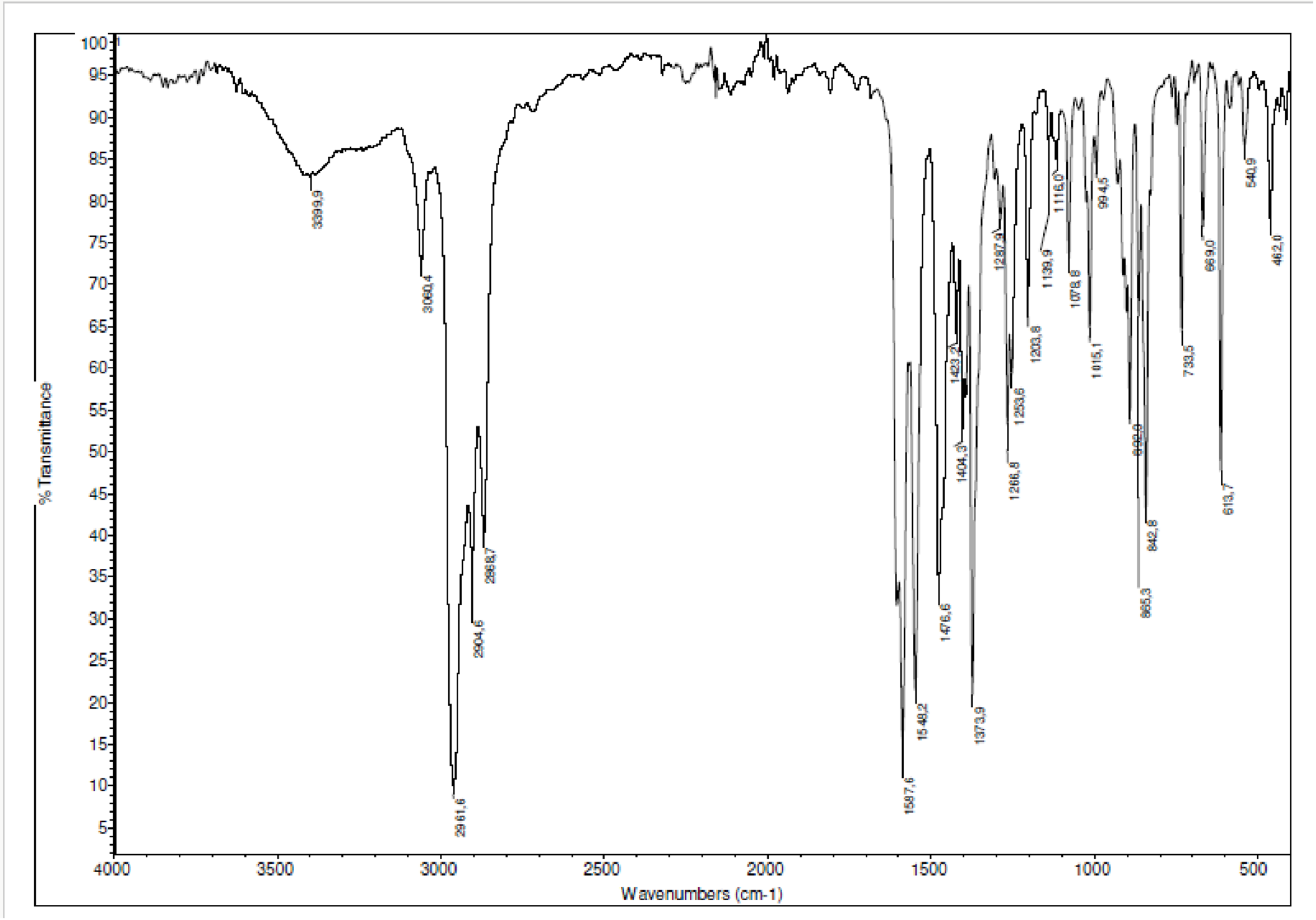

% Transmittance
Wavenumbers (cm-1)
3399,9
3060,4
2961,6
2904,6
2868,7
1587,6
1548,2
1476,6
1423,2
1404,3
1373,9
1287,9
1266,8
1253,6
1203,8
1139,9
1116,0
1078,8
1015,1
994,5
892,9
865,3
842,8
733,5
669,0
613,7
540,9
462,0

c) ^{1}H spectra

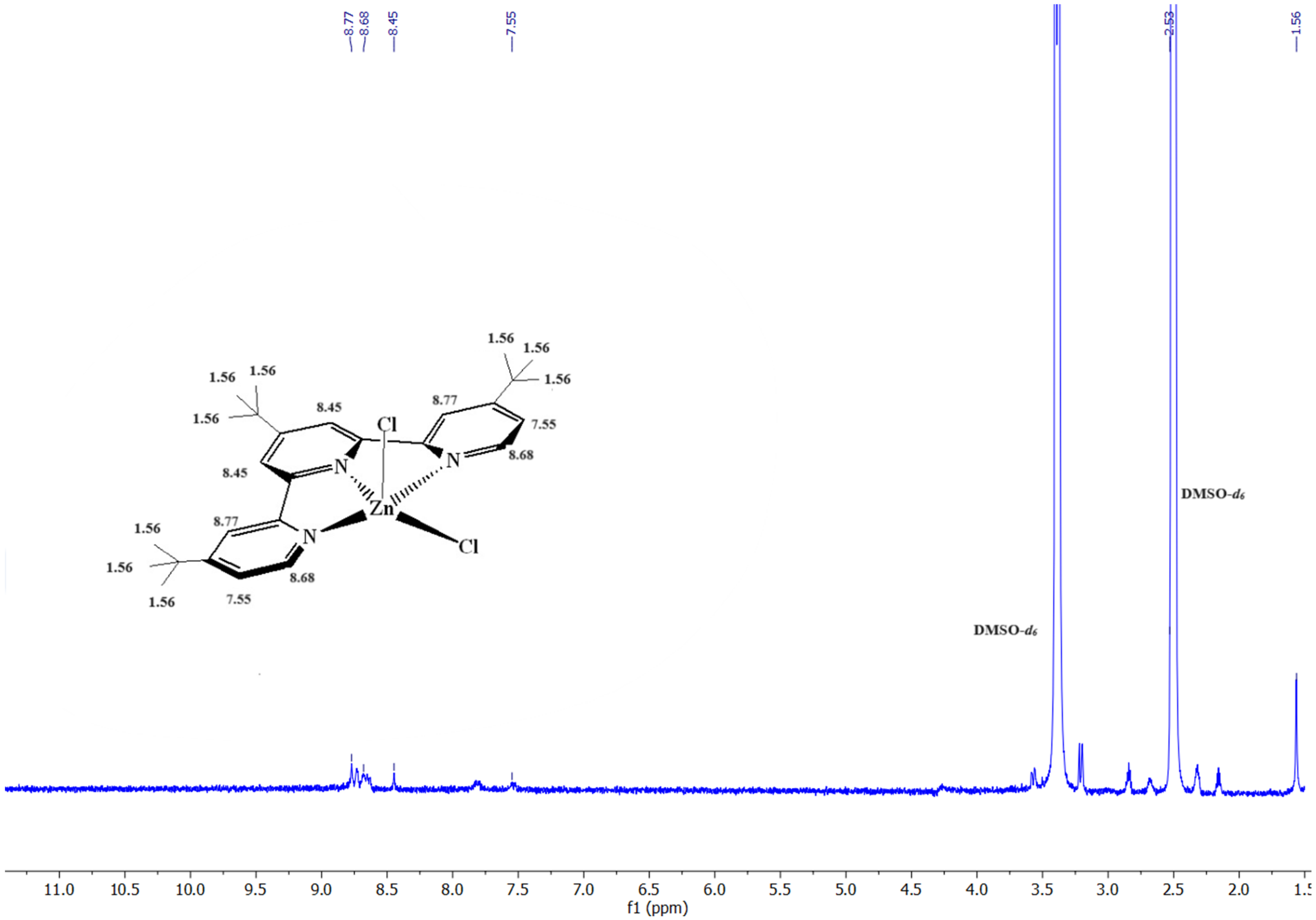

*The peak at 3.38 is due to the moisture present in DMSO-d_6

d) ESI-MS spectra

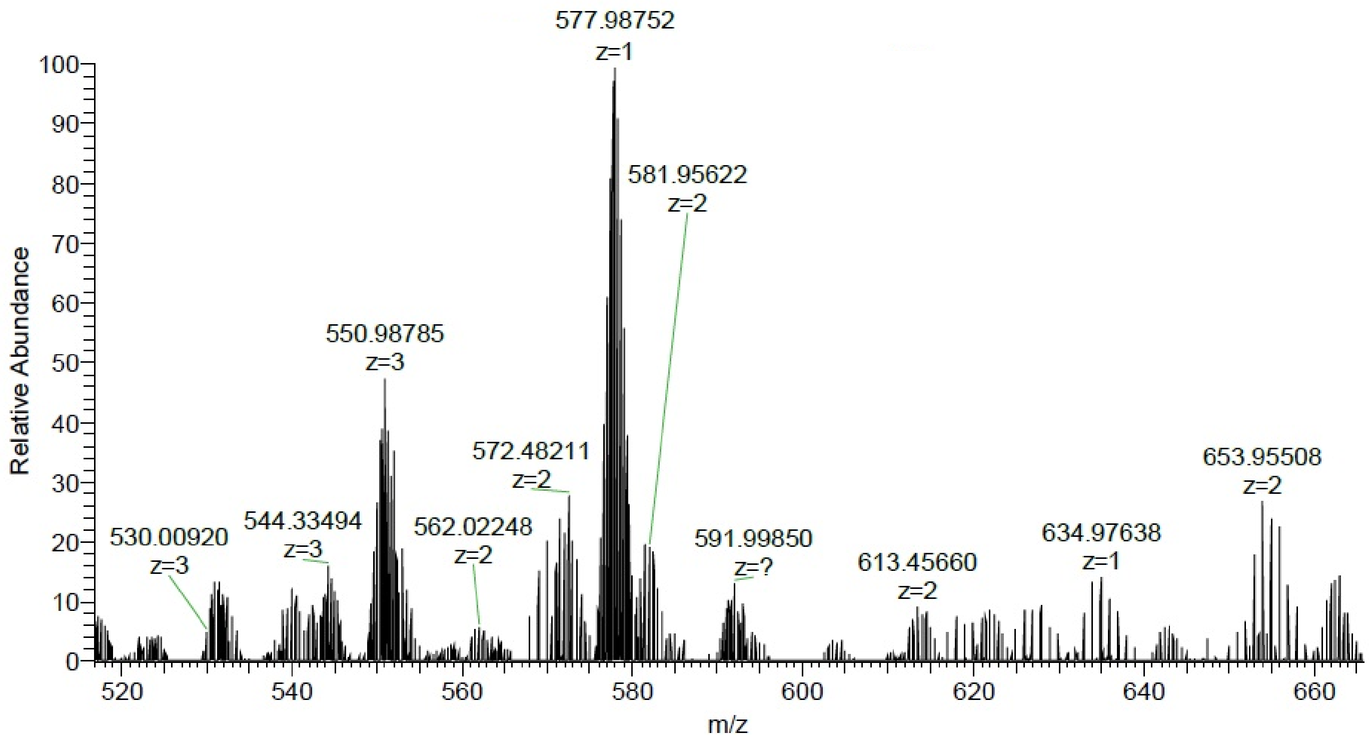

Figure S1. Characterization spectra of [ZnCl$_2$(terpy^{tBu})] complex.

[{*cis*-PtCl$(NH_3)_2$(μ-pyrazine)ZnCl(terpy^{tBu})}]$(ClO_4)_2$

a) UV-Vis

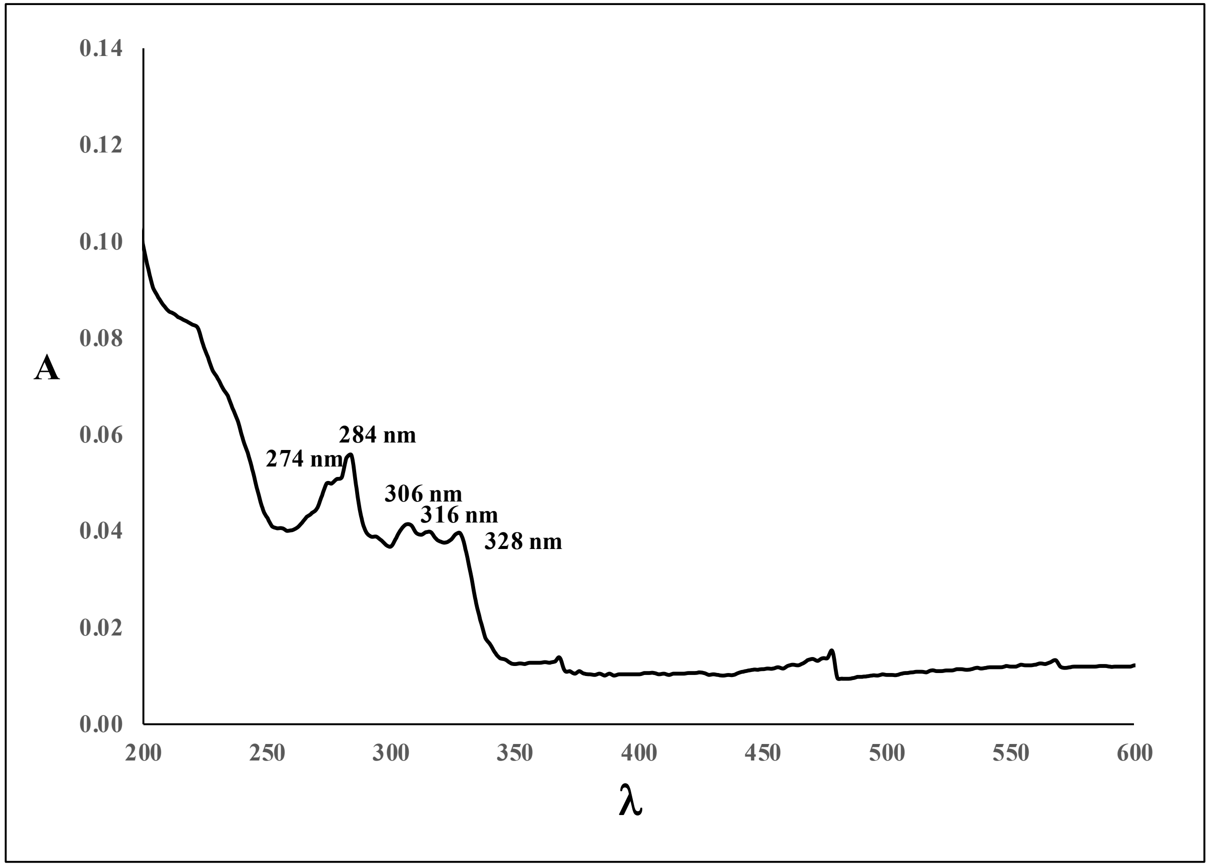

(274, 0.0496) (284, 0.0555) (306, 0.0411) (316, 0. 0396) (328, 0.0393)

b) FT-IR (KBr)

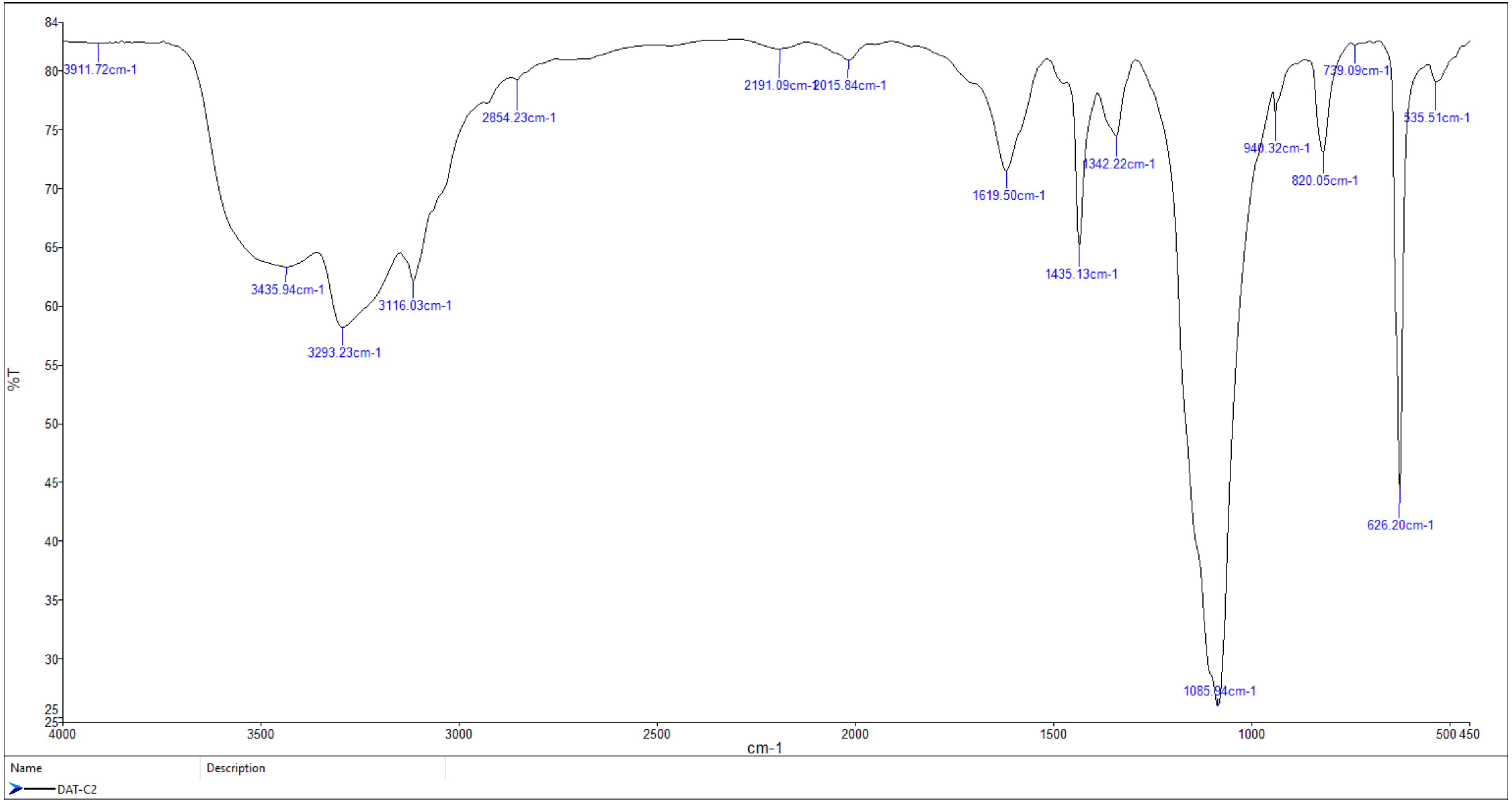

84
80
75
70
65
60
55
50
45
40
35
30
25
%T
4000
3500
3000
2500
2000
1500
1000
500 450
cm-1
3911.72cm-1
3435.94cm-1
3293.23cm-1
3116.03cm-1
2854.23cm-1
2191.09cm-1
2015.84cm-1
1619.50cm-1
1435.13cm-1
1342.22cm-1
1085.94cm-1
940.32cm-1
820.05cm-1
739.09cm-1
626.20cm-1
535.51cm-1
Name
Description
DAT-C2

c) ^{1}H spectra

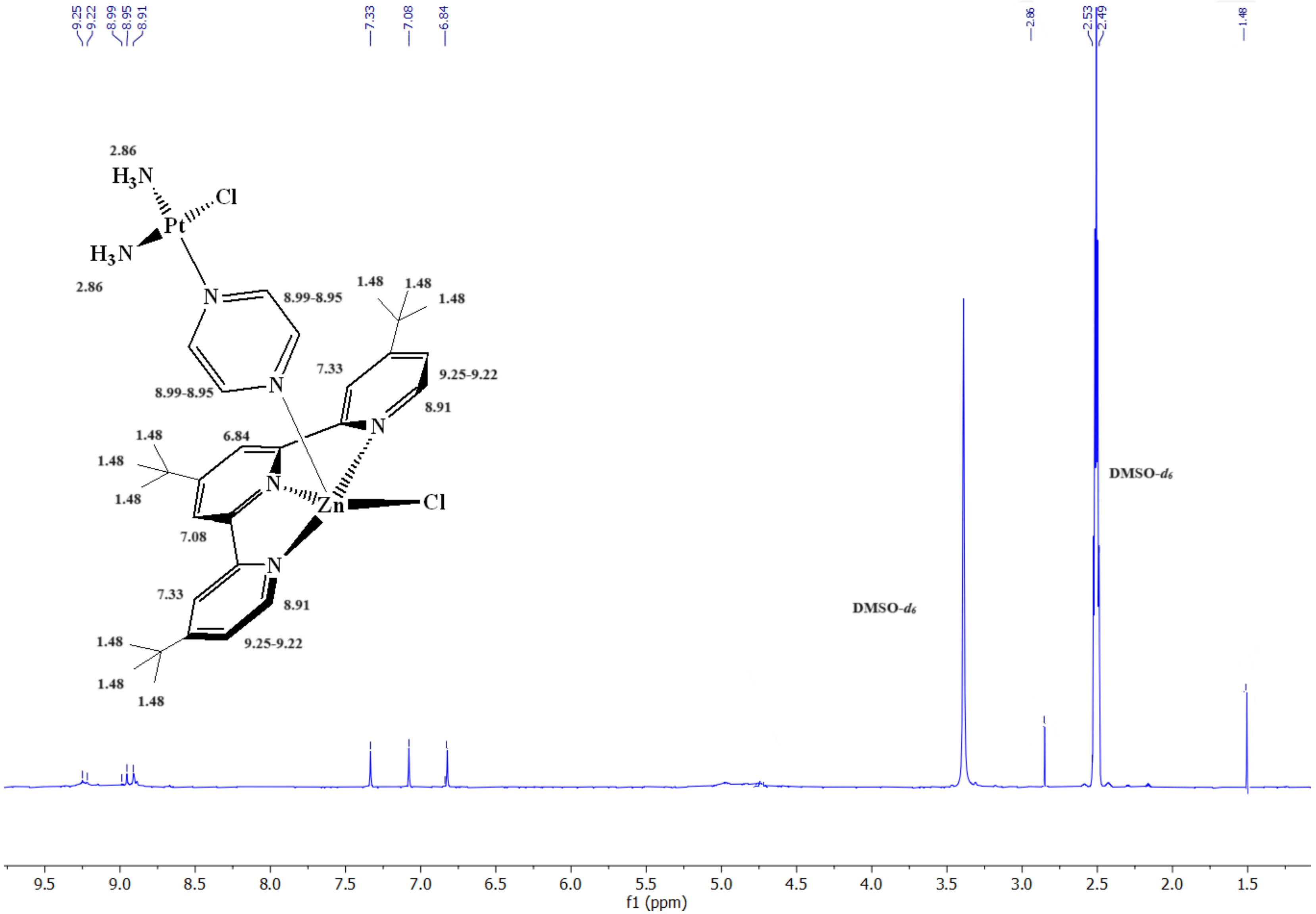

*The peak at 3.38 is due to the moisture present in DMSO-d_6

d) ESI-MS spectra

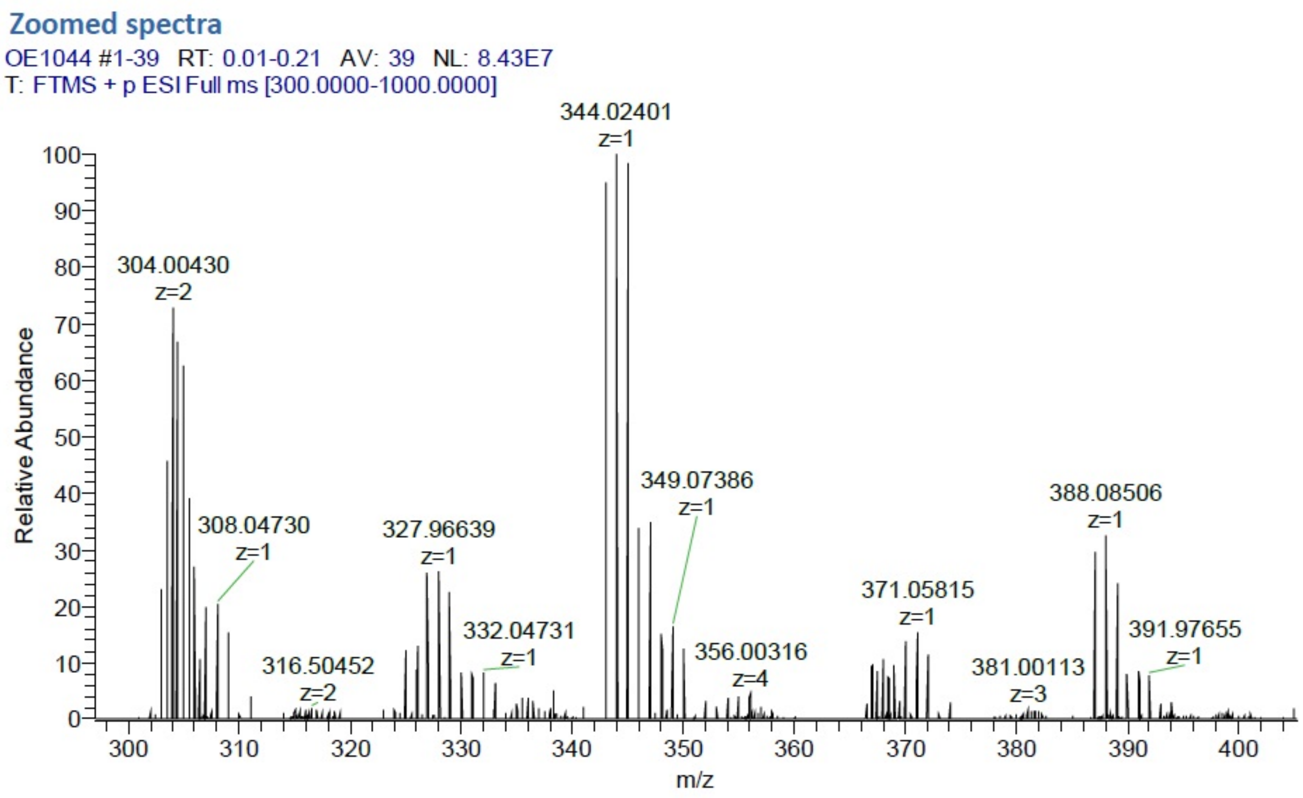

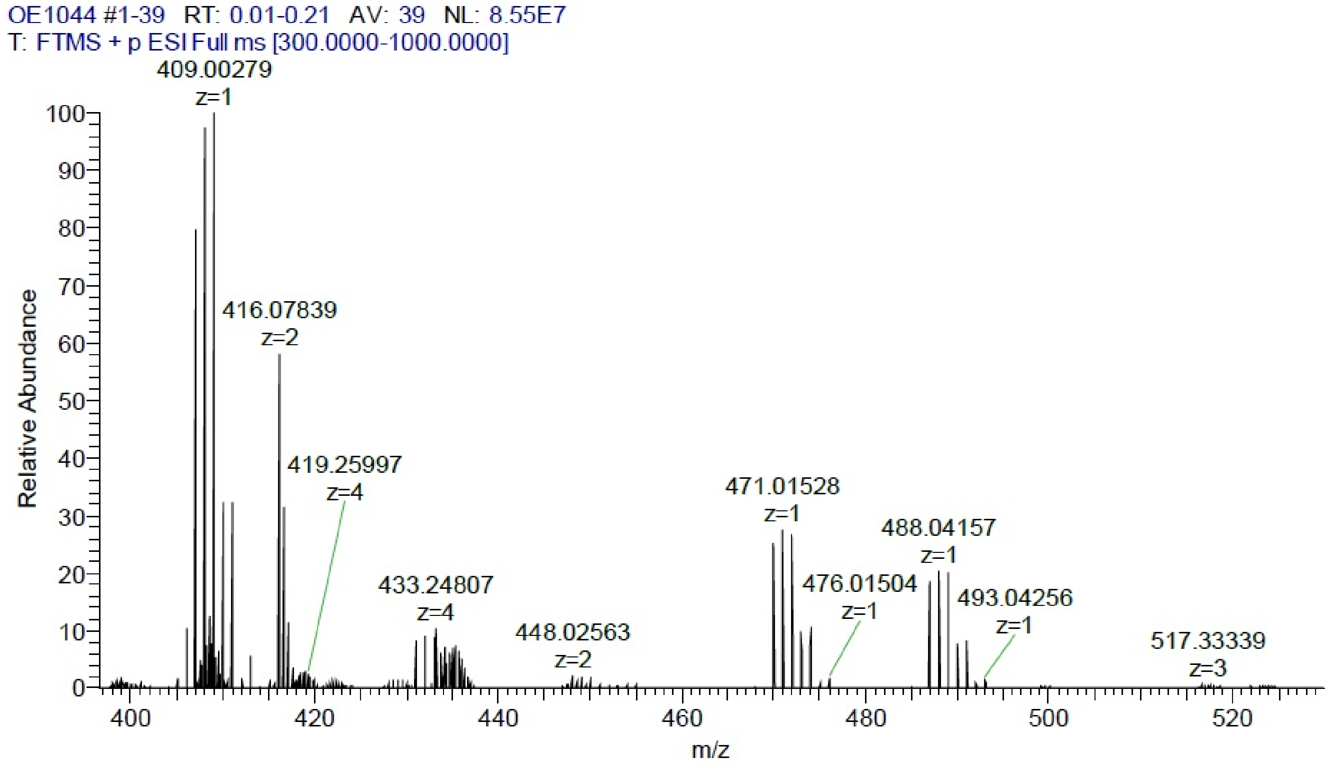

Figure S2. Characterization spectra of [{*cis*-PtCl(NH$_3$)$_2$(μ-pyrazine)ZnCl(terpy^{tBu})}](ClO$_4$)$_2$ complex.

[{*trans*-PtCl(NH_3)$_2$(μ-pyrazine)ZnCl(terpy^{tBu})}](ClO_4)$_2$

a) UV-Vis

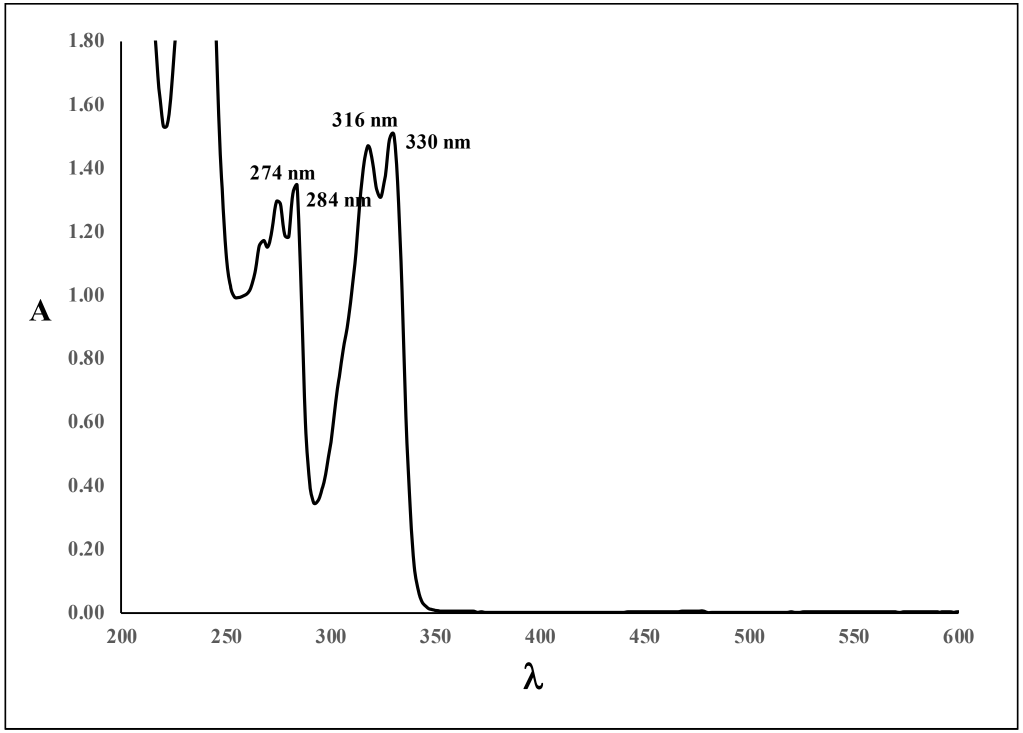

(274, 1.2059) (284, 1.3438) (318, 1.4696) (330, 1.5059)

b) FT-IR (KBr)

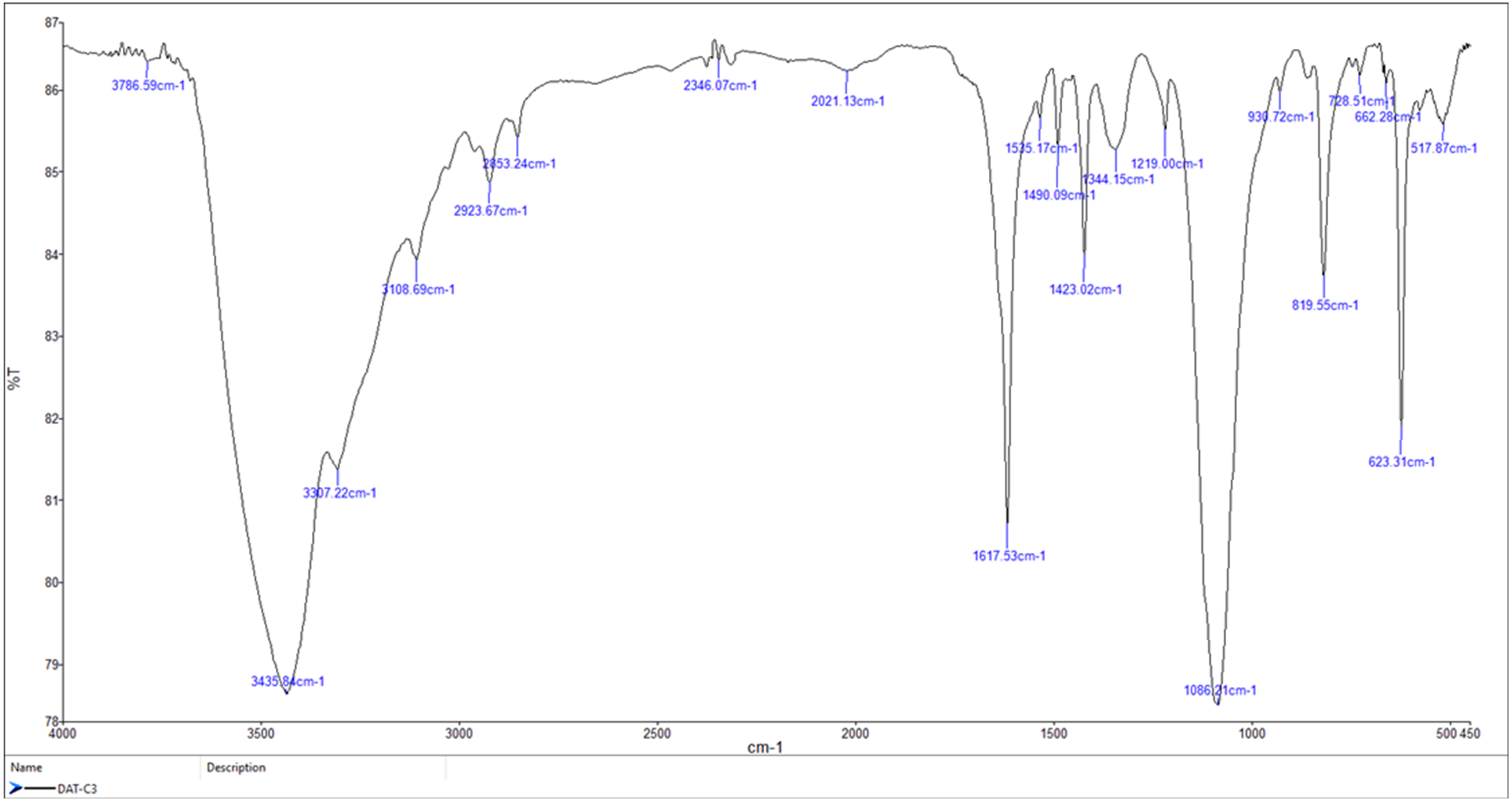

87
86
85
84
83
82
81
80
79
78
%T
3786.59cm-1
3435.84cm-1
3307.22cm-1
3108.69cm-1
2923.67cm-1
2853.24cm-1
2346.07cm-1
2021.13cm-1
1617.53cm-1
1535.17cm-1
1490.09cm-1
1423.02cm-1
1344.15cm-1
1219.00cm-1
1086.21cm-1
930.72cm-1
819.55cm-1
728.51cm-1
662.28cm-1
623.31cm-1
517.87cm-1
4000
3500
3000
2500
2000
1500
1000
500 450
cm-1
Name
Description
DAT-C3

c) ^{1}H spectra

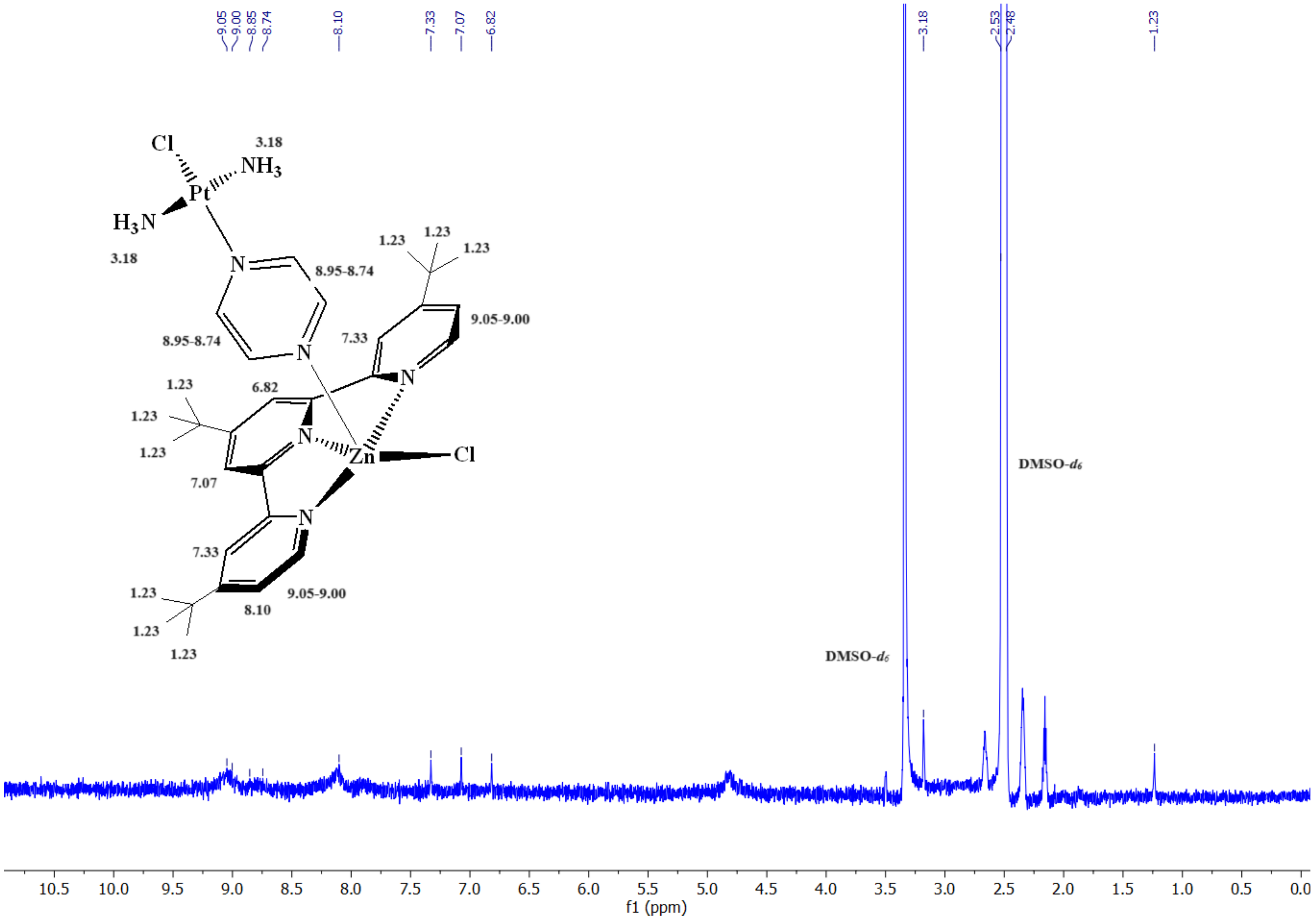

*The peak at 3.38 is due to the moisture present in DMSO-*d*6

Figure S3. Characterization spectra of [{*trans*-PtCl(NH_3)$_2$(μ-pyrazine)ZnCl(terpy^{tBu})}](ClO_4)$_2$ complex.

[{*cis*-PtCl(NH_3)$_2$(μ-4,4'-bipyridyl)ZnCl(terpy^{tBu})}](ClO$_4$)$_2$

a) UV-Vis

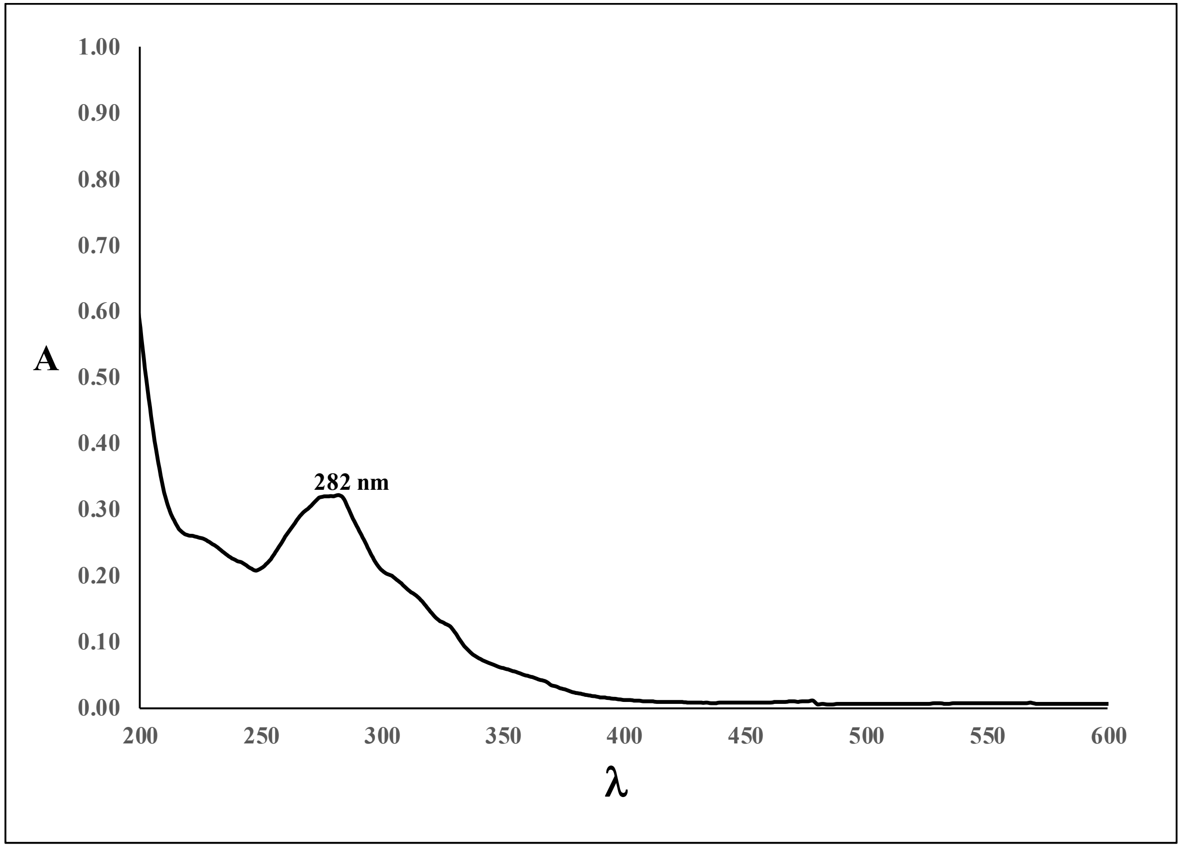

(282, 0.3213)

b) FT-IR (KBr)

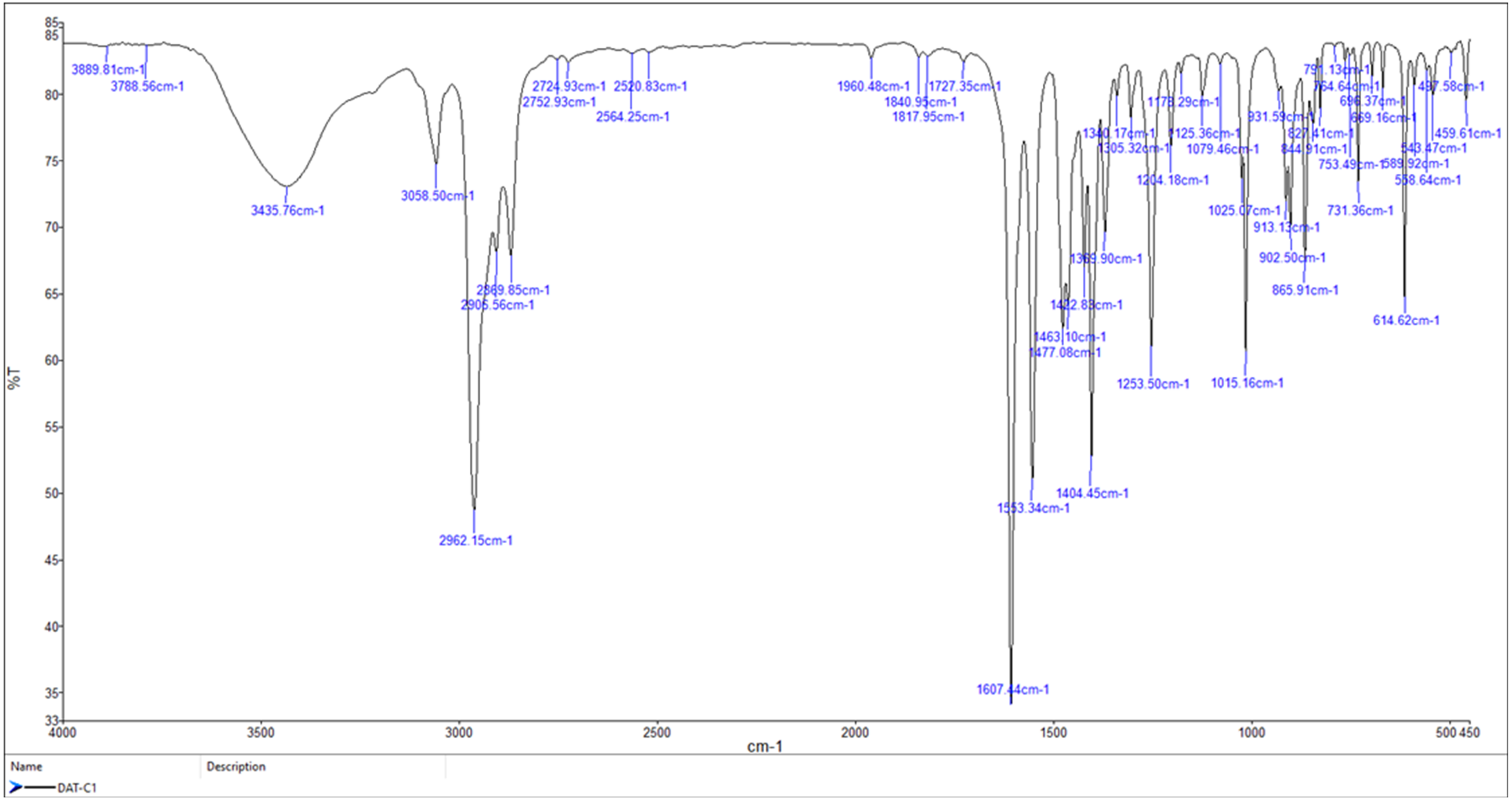

3889.81cm-1
3788.56cm-1
3435.76cm-1
3058.50cm-1
2962.15cm-1
2869.85cm-1
2905.56cm-1
2724.93cm-1
2752.93cm-1
2520.83cm-1
2564.25cm-1
1960.48cm-1
1840.95cm-1
1817.95cm-1
1727.35cm-1
1607.44cm-1
1553.34cm-1
1404.45cm-1
1477.08cm-1
1463.10cm-1
1422.88cm-1
1369.90cm-1
1340.17cm-1
1305.32cm-1
1253.50cm-1
1204.18cm-1
1178.29cm-1
1125.36cm-1
1079.46cm-1
1025.07cm-1
1015.16cm-1
931.59cm-1
913.13cm-1
902.50cm-1
865.91cm-1
844.91cm-1
827.41cm-1
791.13cm-1
764.64cm-1
753.49cm-1
731.36cm-1
696.37cm-1
669.16cm-1
614.62cm-1
589.92cm-1
558.64cm-1
543.47cm-1
497.58cm-1
459.61cm-1
%T
cm-1
Name
Description
DAT-C1

c) ^{1}H spectra

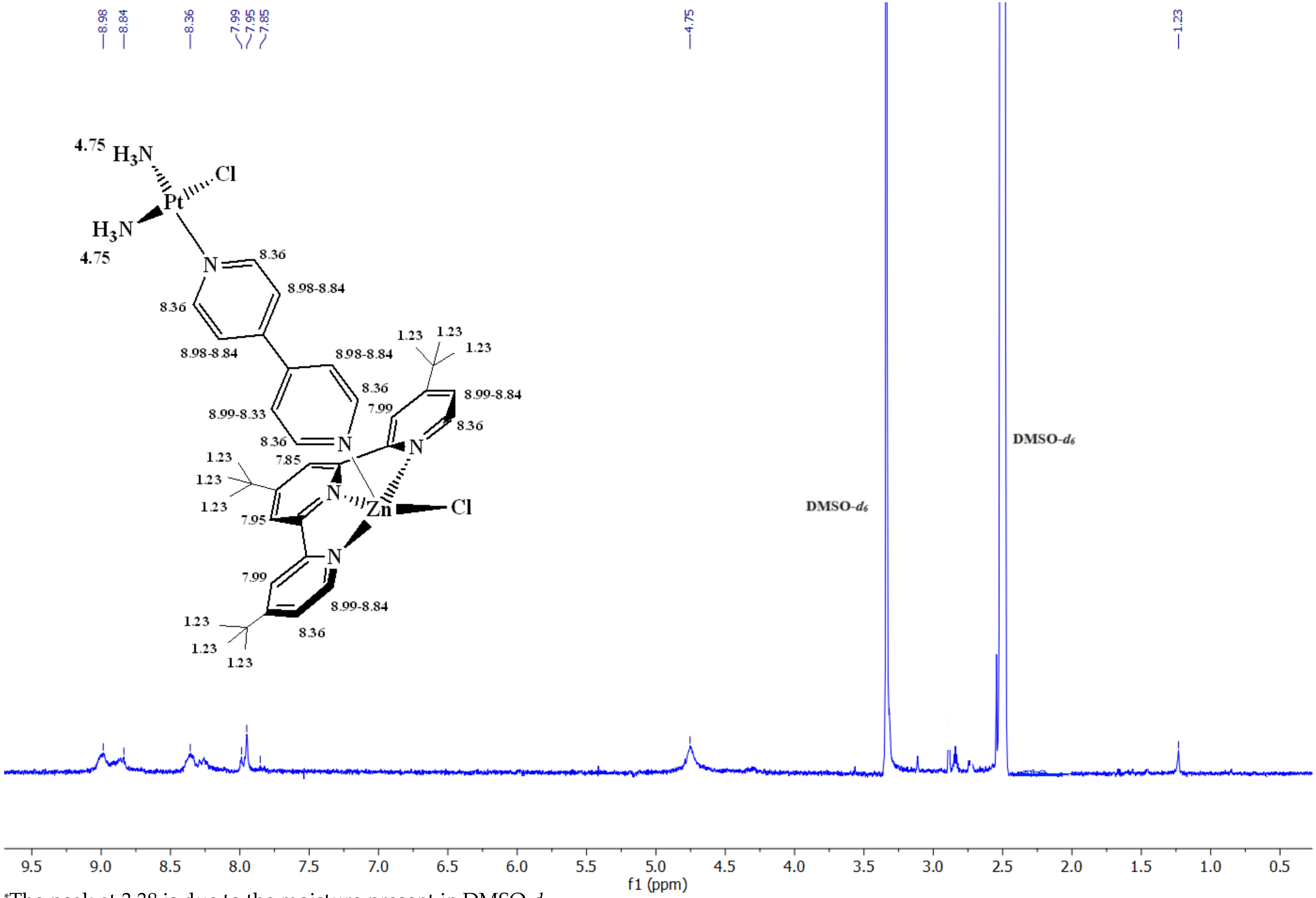

*The peak at 3.38 is due to the moisture present in DMSO-d_6

Figure S4. Characterization spectra of [{*cis*-PtCl(NH_3)$_2$(μ-4,4'- bipyridyl)ZnCl(terpy^{tBu})}](ClO_4)$_2$ complex.

[{*trans*-PtCl(NH3)$_2$(μ-4,4'-bipyridyl)ZnCl(terpy^{tBu})}](ClO$_4$)$_2$

a) UV-Vis

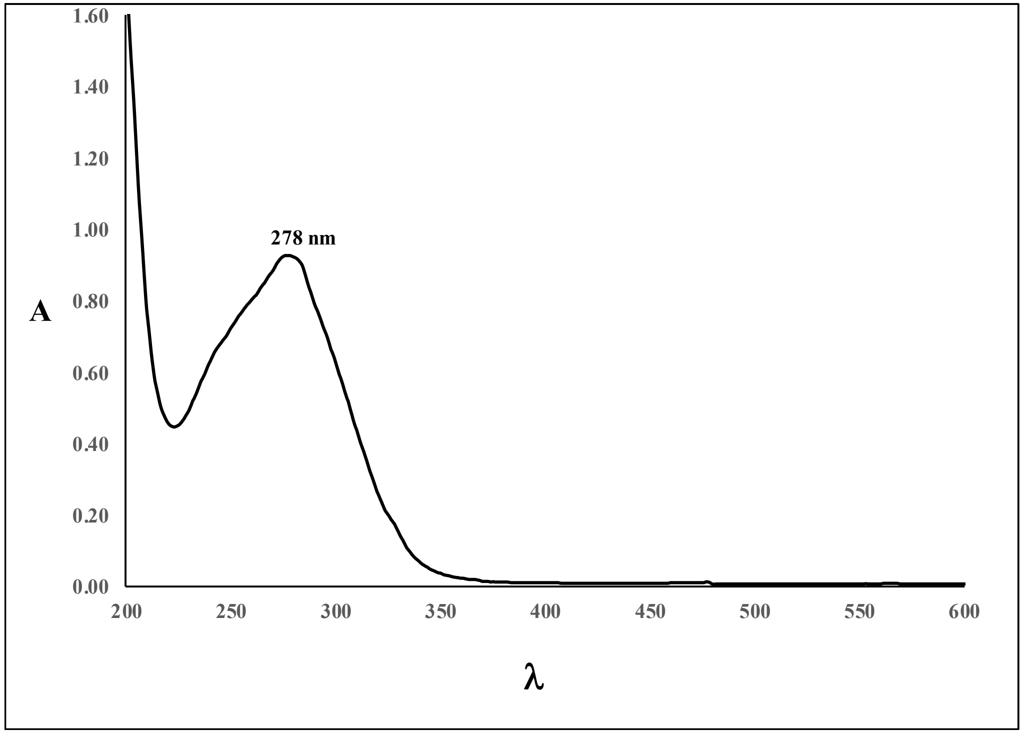

(278, 0.9292)

b) FT-IR (KBr)

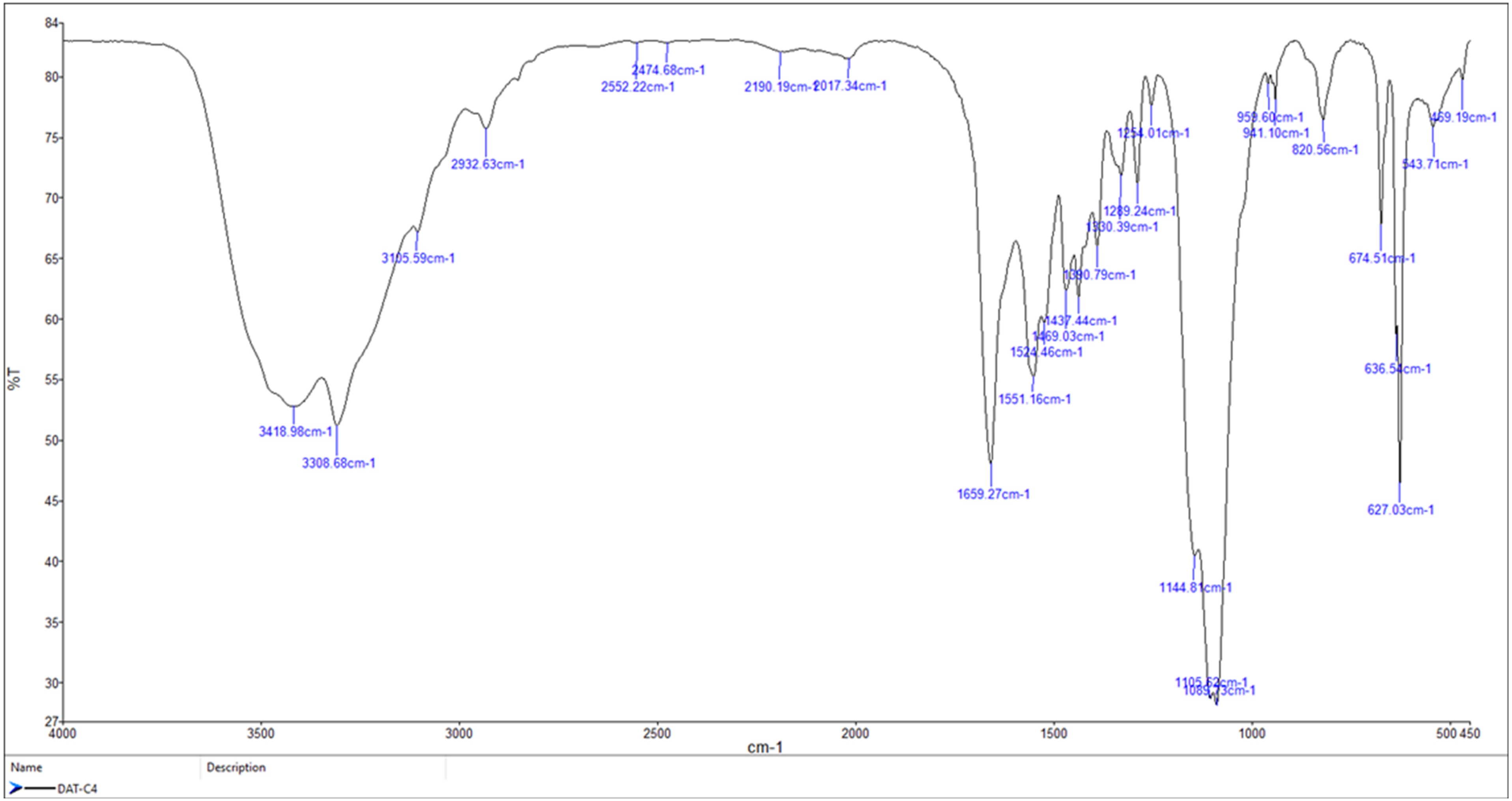

%T
cm-1
3418.98cm-1
3308.68cm-1
3105.59cm-1
2932.63cm-1
2552.22cm-1
2474.68cm-1
2190.19cm-1
2017.34cm-1
1659.27cm-1
1551.16cm-1
1524.46cm-1
1469.03cm-1
1437.44cm-1
1390.79cm-1
1330.39cm-1
1289.24cm-1
1254.01cm-1
1144.81cm-1
1105.62cm-1
1089.73cm-1
959.60cm-1
941.10cm-1
820.56cm-1
674.51cm-1
636.54cm-1
627.03cm-1
543.71cm-1
469.19cm-1
Name
Description
DAT-C4

c) ^{1}H spectra

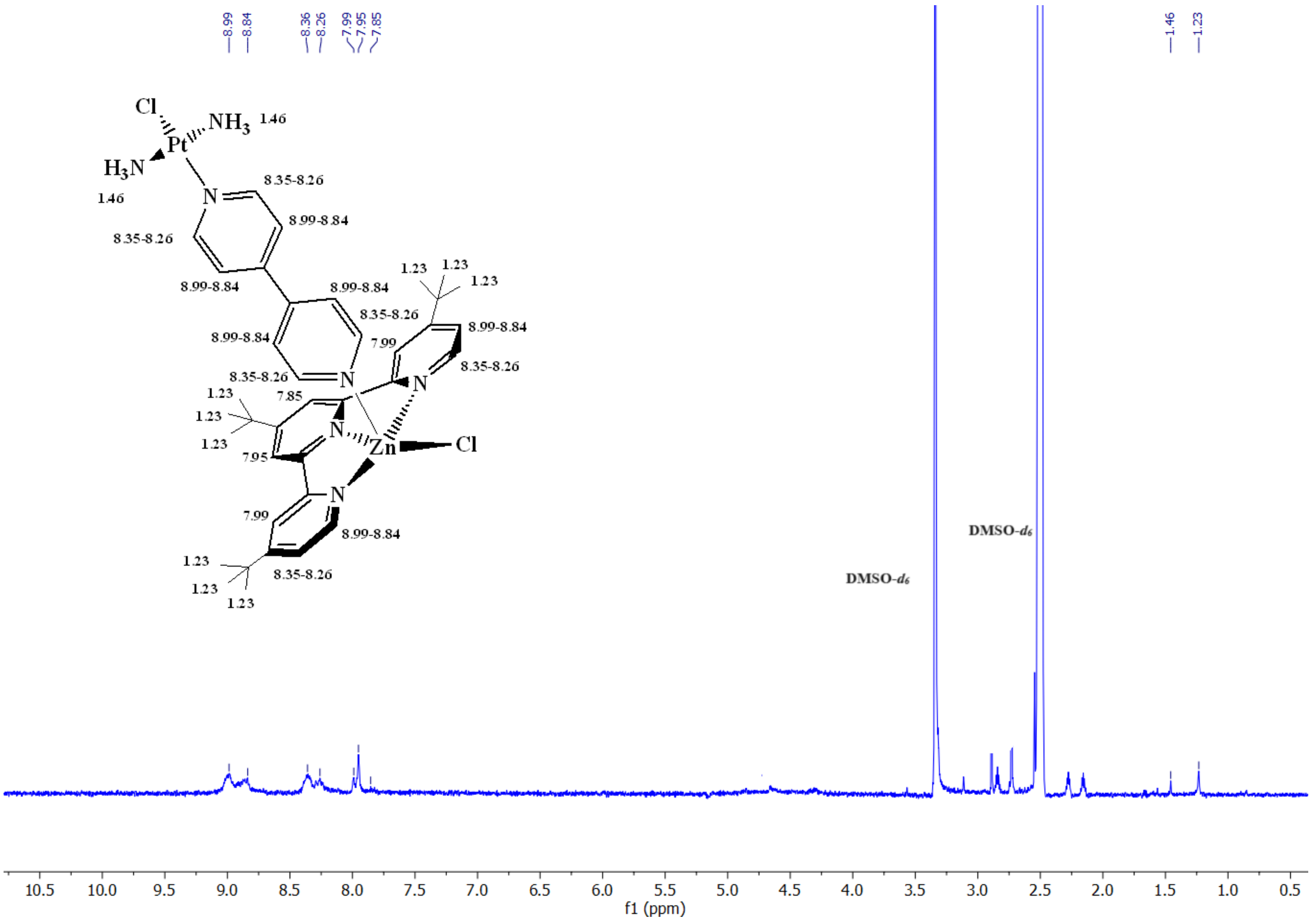

*The peak at 3.38 is due to the moisture present in DMSO-d_6

d) ESI-MS spectra

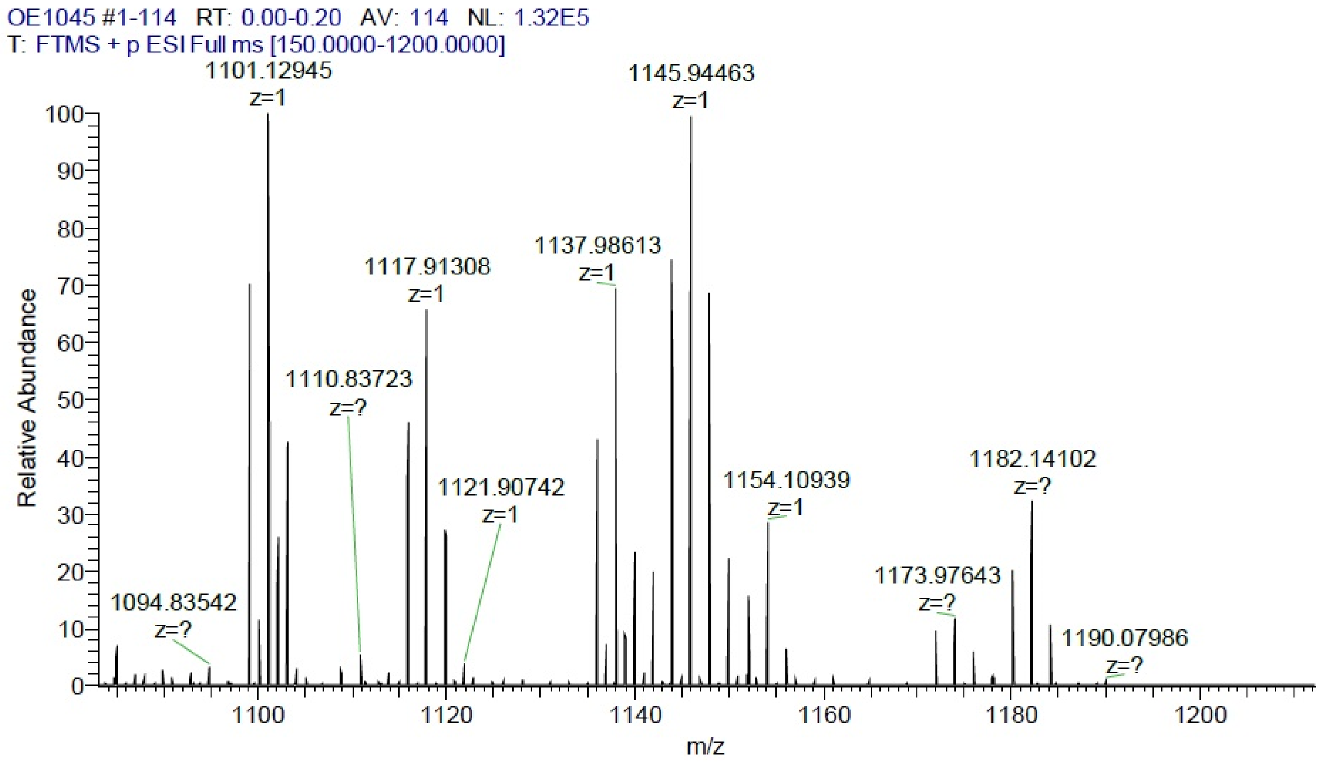

Figure S5. Characterization spectra of[{*trans*-PtCl$(NH_3)_2$(μ-4,4'-bipyridyl)ZnCl(terpy^{tBu})}]$(ClO_4)_2$ complex.

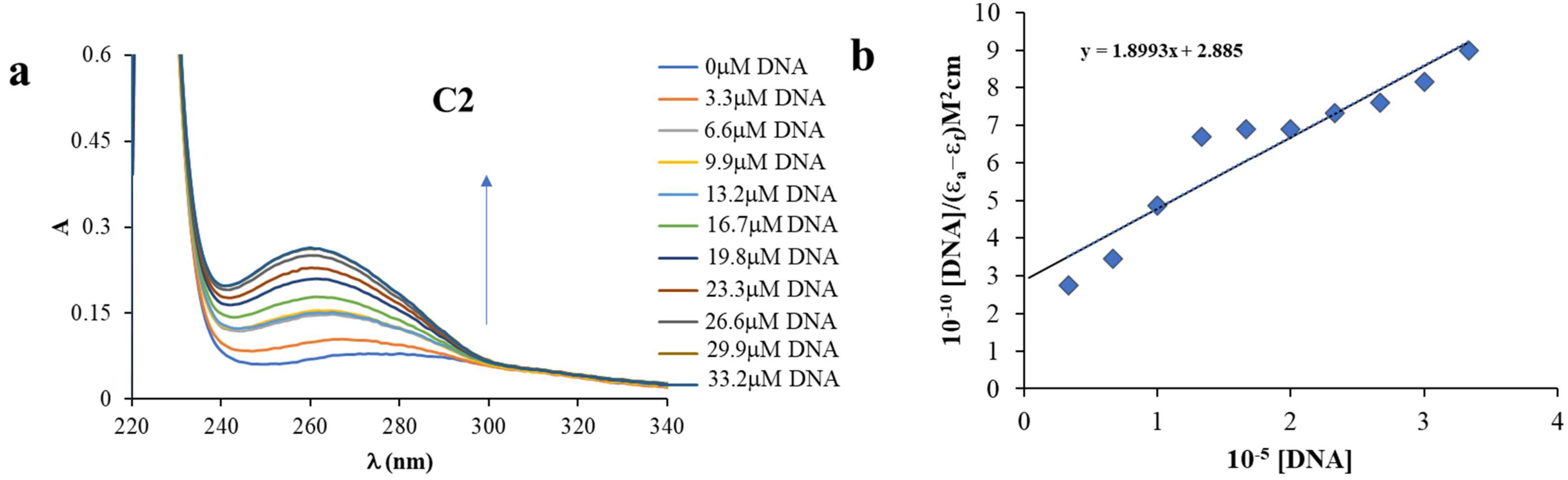

Figure S6. a) Absorption spectra of the examined complex **C2** titration in the presence of CT-DNA (complex concentration was 8 μM, CT-DNA was added up to ratio 5). The arrow shows changes in the spectral band with the increasing CT-DNA concentration. b) Graph showing plots of [CT-DNA]/(εA – εf)] *vs*. [CT-DNA].

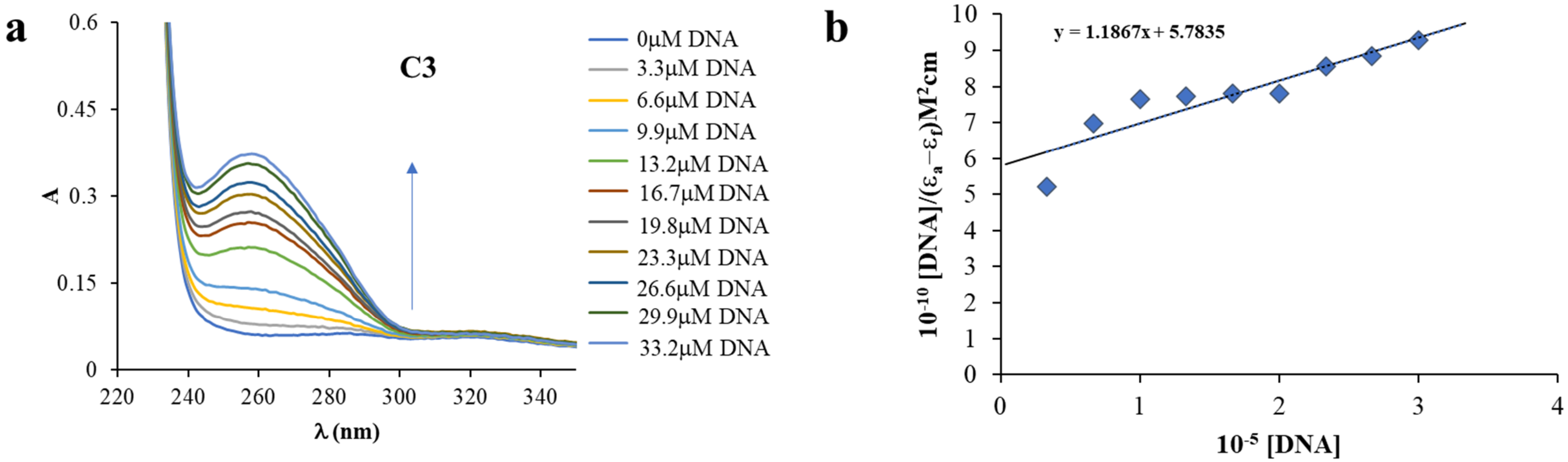

Figure S7. a) Absorption spectra of the examined complex **C3** titration in the presence of CT-DNA (complex concentration was 8 μM, CT-DNA was added up to ratio 5). The arrow shows changes in the spectral band with the increasing CT-DNA concentration. b) Graph showing plots of [CT-DNA]/(εA – εf)] *vs*. [CT-DNA].

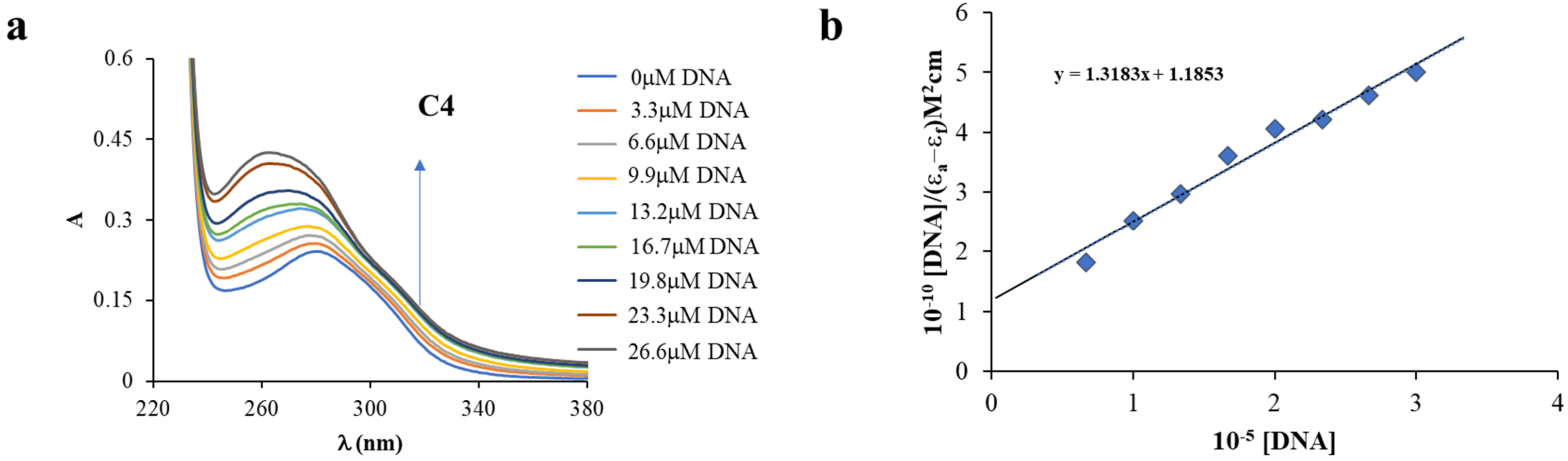

Figure S8. a) Absorption spectra of the examined complex **C4** titration in the presence of CT-DNA (complex concentration was 8 μM, CT-DNA was added up to ratio 5). The arrow shows changes in the spectral band with the increasing CT-DNA concentration. b) Graph showing plots of [CT-DNA]/(εA – εf)] *vs*. [CT-DNA].

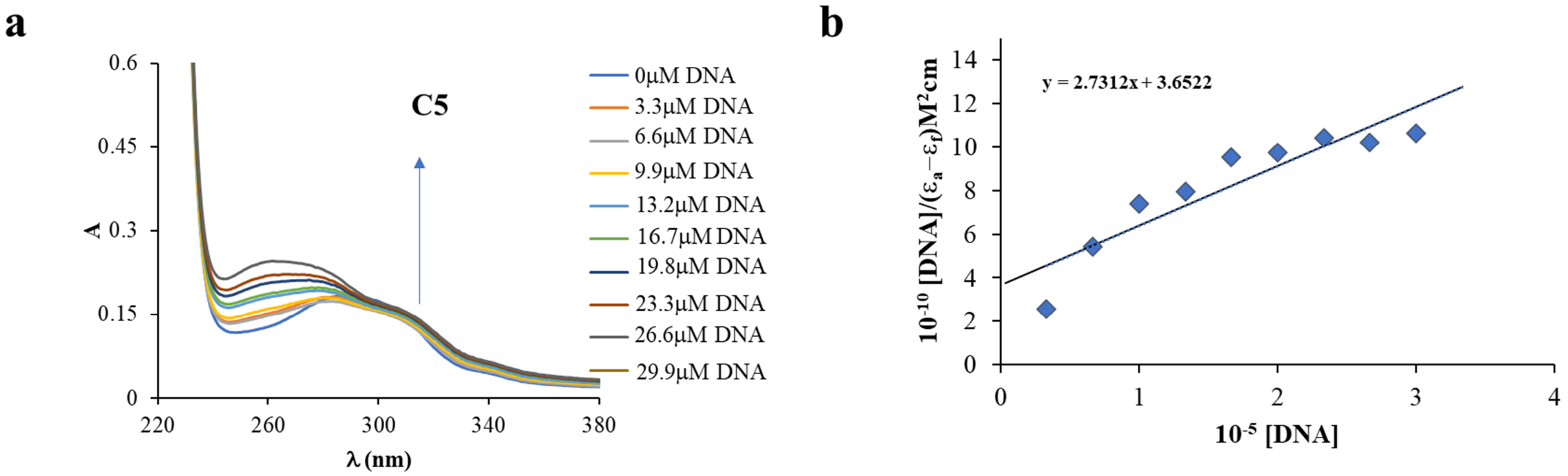

Figure S9. a) Absorption spectra of the examined complex **C5** titration in the presence of CT-DNA (complex concentration was 8 μM, CT-DNA was added up to ratio 5). The arrow shows changes in the spectral band with the increasing CT-DNA concentration. b) Graph showing plots of [CT-DNA]/(εA – εf)] *vs*. [CT-DNA].

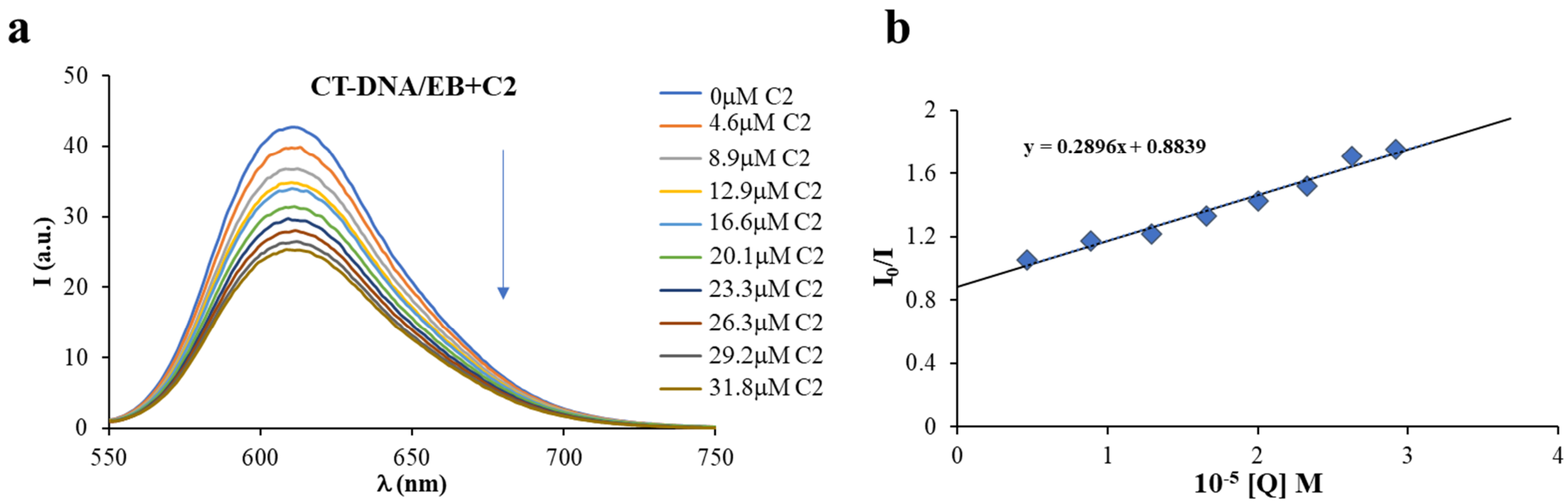

Figure S10. a) Fluorescent titration spectra of EB/CT-DNA solution (ratio EB:CT-DNA was 1:1, 5 μM) in absence and presence of the complex **C2**. The arrow shows changes in the spectral band with the increasing complex concentration (added up to ratio 5). b) Graph showing Stern-Volmer plots for CT-DNA/EB fluorescence titration in presents of the examined complex.

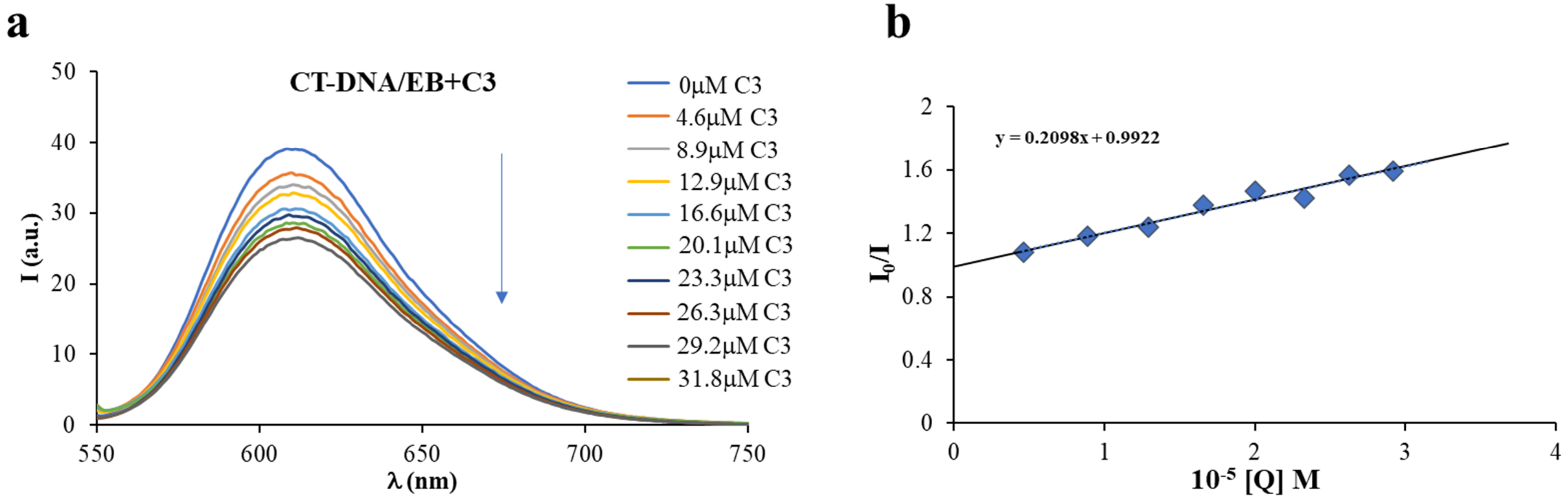

Figure S11. a) Fluorescent titration spectra of EB/CT-DNA solution (ratio EB:CT-DNA was 1:1, 5 μM) in absence and presence of the complex **C3**. The arrow shows changes in the spectral band with the increasing complex concentration (added up to ratio 5). b) Graph showing Stern-Volmer plots for CT-DNA/EB fluorescence titration in presents of the examined complex.

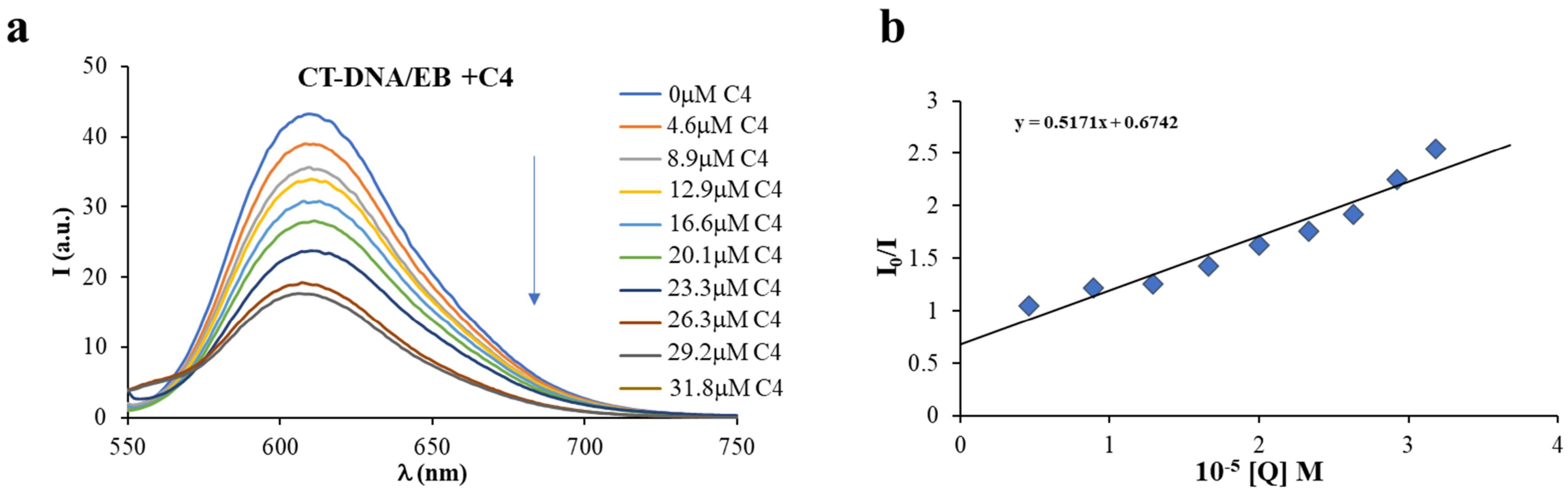

Figure S12. a) Fluorescent titration spectra of EB/CT-DNA solution (ratio EB:CT-DNA was 1:1, 5 μM) in absence and presence of the complex **C4**. The arrow shows changes in the spectral band with the increasing complex concentration (added up to ratio 5). b) Graph showing Stern-Volmer plots for CT-DNA/EB fluorescence titration in presents of the examined complex.

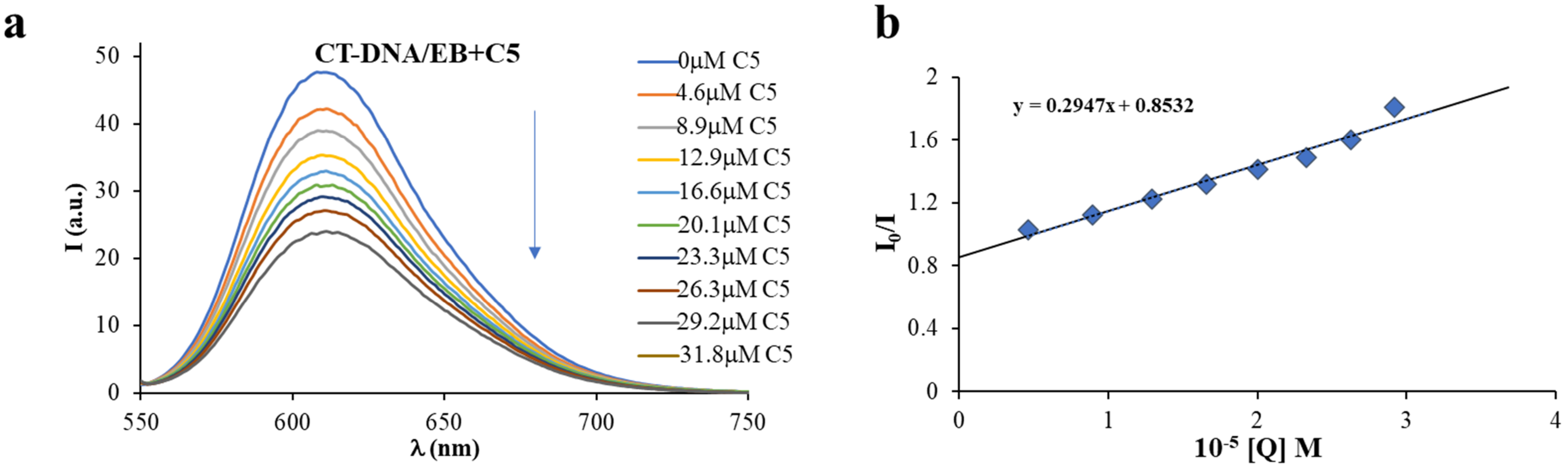

Figure S13. a) Fluorescent titration spectra of EB/CT-DNA solution (ratio EB:CT-DNA was 1:1, 5 µM) in absence and presence of the complex **C5**. The arrow shows changes in the spectral band with the increasing complex concentration (added up to ratio 5). b) Graph showing Stern-Volmer plots for CT-DNA/EB fluorescence titration in presents of the examined complex.

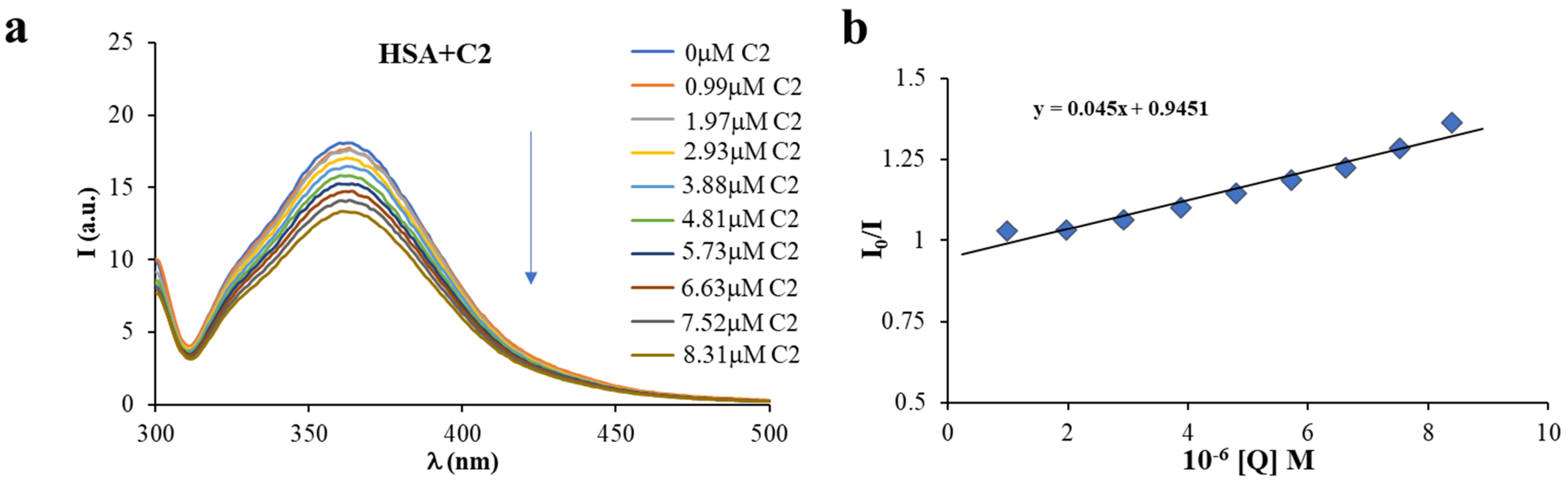

Figure S14. a) Human serum albumin emission spectra in the absence and the presence of the examined complex **C2**, [HSA] = 2 μM, complex up to ratio 5, λ_{ex} = 295 nm. Arrows show changes in intensity after the addition of the growing complex solutions concentration. b) Graph showing the I_0/I dependence of complex concentration. Experimental points are denoted by (♦), with the linear dependence shown by the full lines.

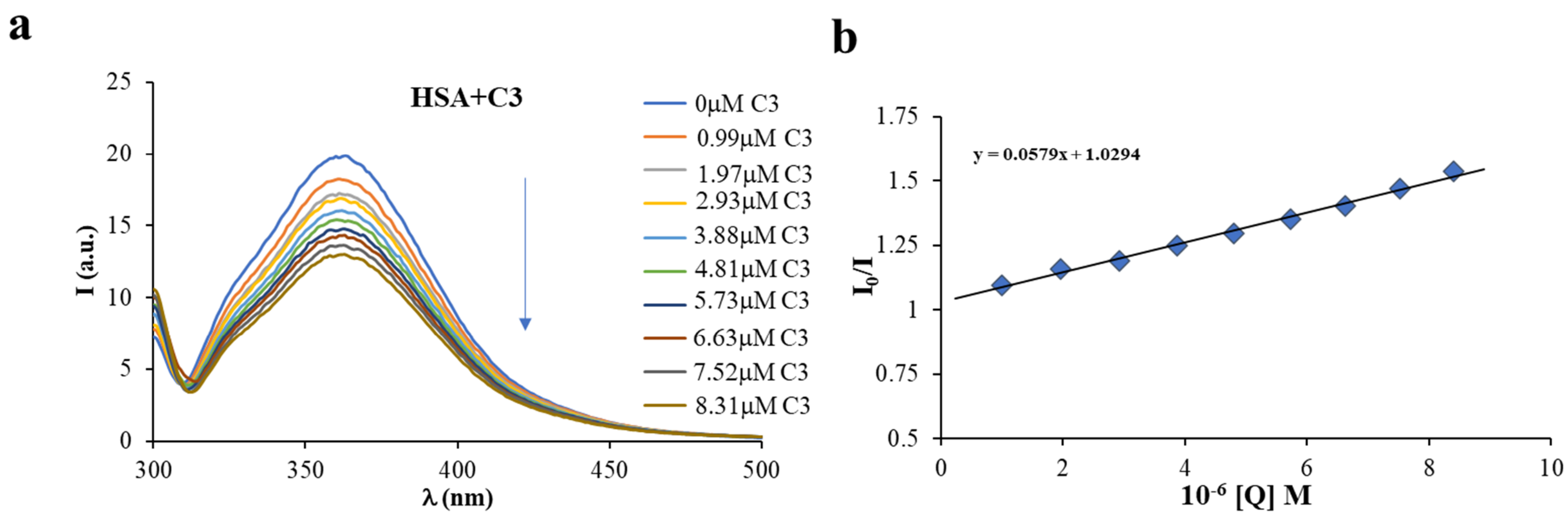

Figure S15. a) Human serum albumin emission spectra in the absence and the presence of the examined complex **C3**, [HSA] = 2 μM, complex up to ratio 5, λ_{ex} = 295 nm. Arrows show changes in intensity after the addition of the growing complex solutions concentration. b) Graph showing the I_0/I dependence of complex concentration. Experimental points are denoted by (♦), with the linear dependence shown by the full lines.

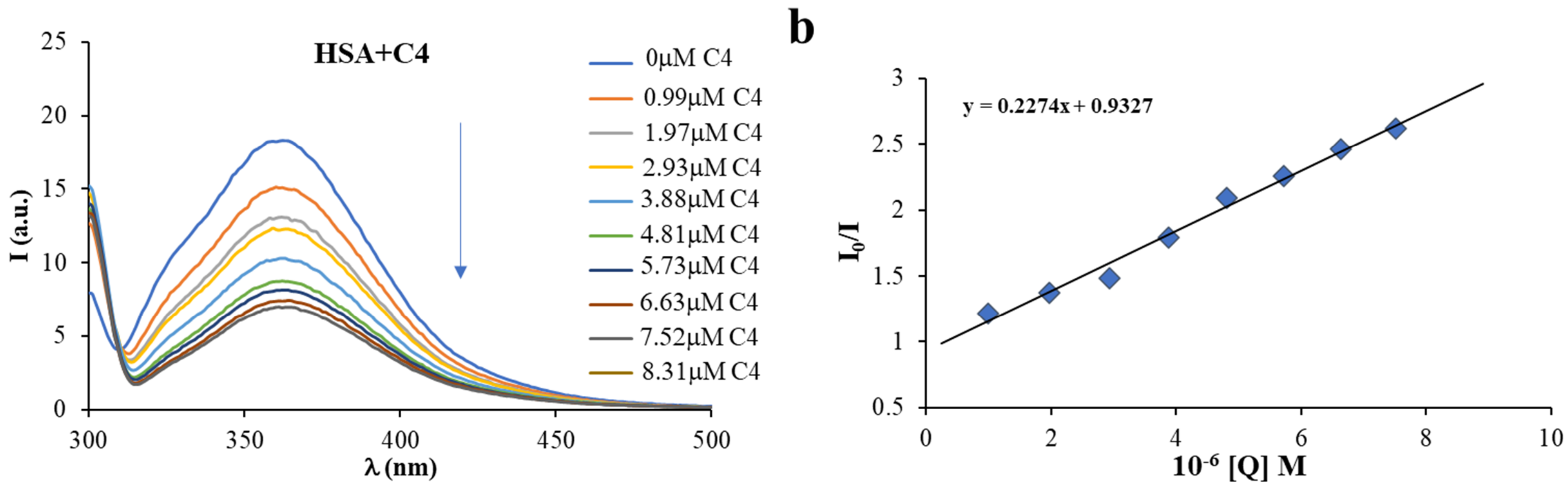

Figure S16. a) Human serum albumin emission spectra in the absence and the presence of the examined complex **C4**, [HSA] = 2 μM, complex up to ratio 5, λ_{ex} = 295 nm. Arrows show changes in intensity after the addition of the growing complex solutions concentration. b) Graph showing the I_0/I dependence of complex concentration. Experimental points are denoted by (♦), with the linear dependence shown by the full lines.

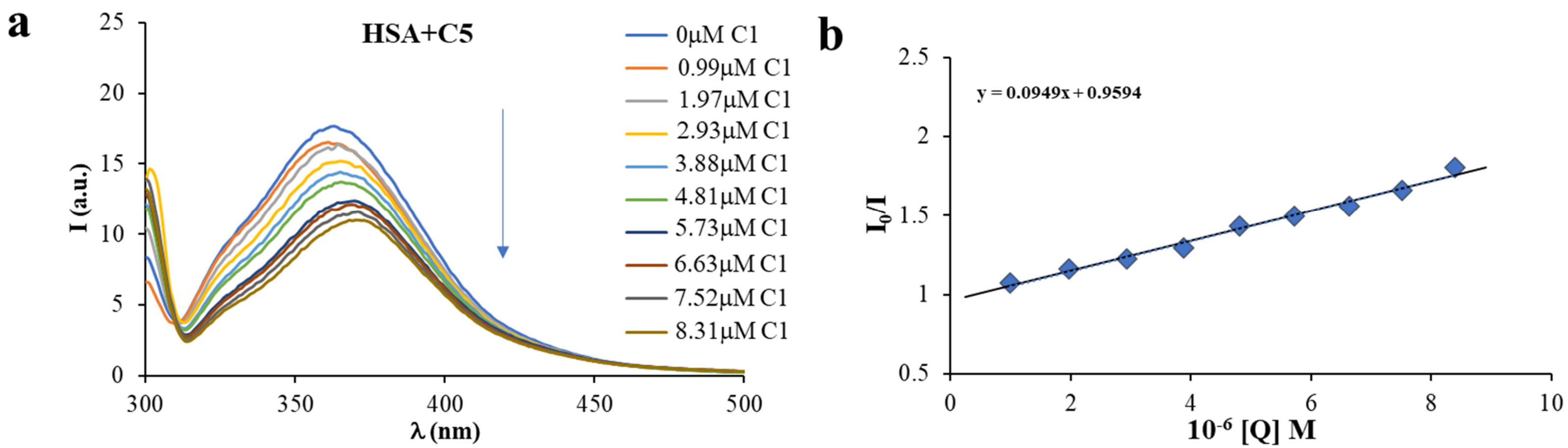

Figure S17. a) Human serum albumin emission spectra in the absence and the presence of the examined complex **C5**, [HSA] = 2 µM, complex up to ratio 5, λ_{ex} = 295 nm. Arrows show changes in intensity after the addition of the growing complex solutions concentration. b) Graph showing the I_0/I dependence of complex concentration. Experimental points are denoted by (♦), with the linear dependence shown by the full lines.

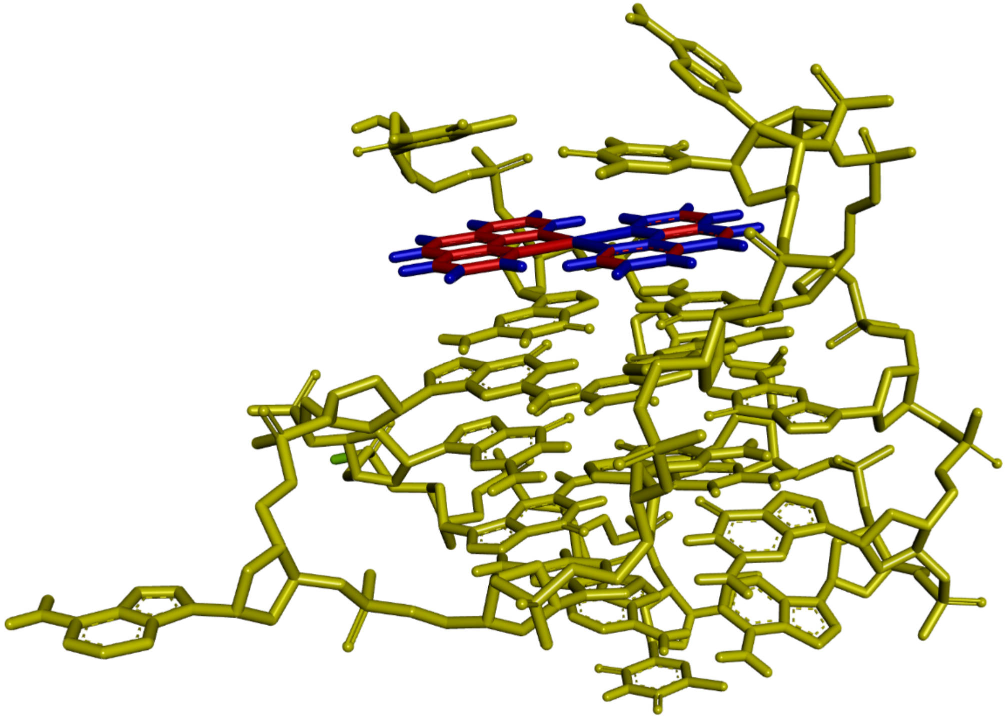

Figure S18. Graphical interpretation of validation docking procedure. DNA molecule is represented in yellow color. Crystallized ligand shown in red color representation. Re-docked pose of the ligand shown in blue color representation.

In order to examine the correctness of the utilized docking procedure, we decided to perform its validation. The Bis(1,10-phenanthroline)platinum(II) was removed from the original Protein Data Bank entry (PDB ID: 7DJW). The compound was re-docked onto the previously created grid file by Autodock 4.2 [44] software equipped with the graphical user interface (GUI) Auto-DockTools (ADT 1.5.6rc3) [15]. The best docking pose was superimposed on the original RCSB PDB entry with Discovery Studio Visualizer 3.5.0 Accelrys Software Inc. [42] and the RMSD score was calculated (0.912 Å).
